# Supplementary material for: Assessing the Transmissibility of the New SARS-CoV-2 Variants: From Delta to Omicron
Source: Vaccines (Basel). 2022 Mar 24;10(4):496. doi: 10.3390/vaccines10040496 (PMC9026126; doi:10.3390/vaccines10040496)
Supplement: Supplementary file 1 [file vaccines-10-00496-s001.zip › vaccines-1615633-supplementary.pdf]

Supplementary Material

1. Time Interval of VOC

For each VOC, starting date of the time interval is defined as when a significant trend of increasing is observed. For B.1.617.2, the earliest one was submitted to GISAID on 2021-02-18 but the sample collected on 2020-09-07. There are about ten B.1.617.2 records on GISAID collected on 2021-02-21, then the number increased dramatically. The time interval for B.1.617.2 is defined to be 2021-02-21 to 2021-07-17. At the end of July, the proportion of B.1.617.2 starts to decrease, and therefore we keep only the early development stage of it.

On the other hand, AY.4 appears in May followed by a significant expansion. We therefore select 2021-05-30 to 2021-07-24 as the time interval for AY.4.

For Omicron, it appears firstly in early November in Africa, but the data in Africa is not very reliable due to the practical limitation. Since an Omicron variant, BA.1, appears in UK in late November, there have been over 8,000 Omicron variants with complete genomes on GISAID so far. Considering the high index of UK in Table 1, we select the Omicron data in UK with the time interval as 2021-11-28 to 2021-12-10.

2. Data Details and Adjustment

Information about the variants were retrieved from GISAID, including the metadata and all viral sequences of SARS-CoV-2 uploaded to this worldwide platform as of 2021-12-19. Metadata gives a detailed description of each record, including the age, gender of host, the sampled location, collection date, and submission date, the Pango lineage.

Another reliable data source is the daily report from the Johns Hopkins University Center for Systems Science and Engineering (JHU CSSE) (https://github.com/CSSEGISandData/COVID-19). We collect epidemic data including daily confirmed, recovered, and death cases on country level around the world from JHU data. We adjust for the missing information of actual size of different lineages of SARS-CoV-2, based on the assumption that the proportion of variants in overall population in each country is identical to those uploaded to GISAID. Combining the statistics from two ends, an index is defined as the ratio of submitted sequences on GISAID to the daily report cases on JHU CSSE during a specific time period, describing data transparency for each country.

In addition, data on NPIs and vaccination are downloaded from the website (https://ourworldindata.org/policy-responses-covid) [1,2]. The data included scores from 0 to 7 on restriction of 13 NPIs, including school closures, workplace closures, cancellation of public events, restrictions on public gatherings, stay-at-home restrictions, face coverings, public information campaigns, public transport, international travel controls, testing policy, contact tracing, income support, debt and contract relief. Additionally, we add vaccination policy as a variable in our model.

3. Bayesian Coalescent Phylo-Dynamic Analysis

For each VOC and its pre-dominant variant in a specific country, 100 genomes of VOCs (B.1.617.2 or AY.4) and 100 of its pre-dominant variants are selected, with proportion to the number of records collected in each month.

The clock rates of VOC and its pre-dominant variant are estimated using BEAST v1.10.4 [3] on the 100 genome sequences of VOC and 100 of its pre-dominant variants. The BEAST analysis employed the Skygrid non-parametric model for demographic history [4], with the same parameters by Volz and colleagues [5], except for a higher number of states as one billion in contrast to 100 million to get a more robust result. The minimum effective sample size was larger than 100 in all parameters for each run.

4. Statistical Analysis

Among the five countries, we sequentially completed the following analysis to show the relative advantage of the VOC against the pre-dominant variant for each country, where VOCs includes B.1.617.2 and AY.4 with their own time period. The ratio of the reproductive numbers of VOC to the pre-dominant variant is estimated to indicate the relative advantage of VOC. And the estimation of reproductive number is based on two parameters, the additive difference of additive growth rate between VOC and pre-dominant, and the clock rate estimated in Section 3.5.

To estimate the difference of additive growth rate for VOC and pre-dominant variant, we construct a well-structure Bayesian framework. We denote the additive difference in growth rate for the VOC against pre-dominant variant as $\rho(t)$, a time-varying continuous index. It indicates the relative advantage of transmission for the variant over the other. We improve the hierarchical Bayesian model in research by Volz and colleagues [5] by considering the correlation between countries, rather than treating them independently. We also add variables about the policy implemented during the period in each country into the model. As stated by Guner and colleagues [6], government interventions on restricting the public event played an important role in controlling the epidemic transmission. However, few researches were conducted to compare the effectiveness of policies on the spread of different variants. Our model considers 13 different stringencies [1,2] as potential influential factors affecting the additive difference growth rate.

Then we estimated the effective size of each variant in country-level with BEAST, based on the viral sequence information and corresponding collection date only. With estimated effective size, the growth rate of each variant can be estimated. The detailed steps are shown as follows: (1) We perform the Bayesian coalescent phylo-dynamic analysis with BEAST v1.10.4 with a sub-sample of 100 sequences each from the VOC and the pre-dominant variant. The hyper-parameters are selected identically as in the research by Volz and colleagues [5]. Bayesian Skygrid model is selected for tree analysis with a total of 42 time-intervals. We assume the substitution as a Jukes-Cantor model and it follows a strict clock model. The other parameters are left as default. For each variant, we run a MCMC for one billion iterations and thinned to 10,000 samples. 10% of the left samples are removed as the "burn-in" period. Effective size for each variant is summarized with Tracer v1.7.2 for two independent trials to compare VOC and its pre-dominant variant. We obtained the minimum effective sample size (ESS) for B.1.617.2 and AY.4 as 161 and 99, respectively. Eventually we can obtain the estimation and confidence interval of clock rate for each variant. (2) We conduct the estimation of maximum likelihood tree in IQtree v2.1.3 assuming a HKY model for substitution. (3) We utilize existing packages "treedater" to estimate the time-scaled phylogeny, with prior of clock rate obtained with BEAST above and estimated maximum likelihood trees. (4) Finally, we use "mlesky" model to fit estimated dated trees and obtained an estimation of effective size with 95% confidence interval with bootstrapping. The time-varying growth rate for each variant of our interest is estimated as the empirical growth rate of effective size.

Finally, we conduct estimation of time-varying reproductive number R_0 for VOC and its pre-dominant variant in each country.

To predict when novel variant will become VOC based on early collected data, we conducted simulation with data on Alpha variants in the United Kingdom, Delta variants in India and Omicron variant in United Kingdom, respectively. First, we estimated the posterior distribution of parameters given in the logistic linear model under a Bayesian framework. With estimated posterior, we simulated the growth curve of the proportion of novel variants for 10,000 independent trials. For each trial, we recorded the date when the proportion of variant exceeding 50%, which can be regarded as a random sample of date

to become VOC. The 90% confidence interval was estimated as the 5% of randomly samples for lower bound and 95% for upper bound. We selected the median of sample as the predicted values due to asymmetry of samples.

5. Sensitivity Analysis

We conduct the sensitivity analysis on the violation of the assumption that the JHU daily report is accurate on the total cases. We assume the actual daily cases in each country are reported cases collected from JHU daily report multiplied with a fixed factor and we let the factor be 0.5, 1, and 2 respectively. The following results in Tables S1–S3 are the estimated additive difference in the growth rate of the Omicron variant in the UK from 2021-11-28 to 2022-12-10, a total of 12 days. We find that there are only minor differences in the estimation in each case.

Table S1. The estimated additive difference in growth rate when daily cases is half as JHU daily report (factor = 0.5).

	**2.50%**	**25%**	**50%**	**75%**	**97.50%**
2021-11-28	0.21	0.37	0.46	0.54	0.72
2021-11-29	0.15	0.29	0.36	0.43	0.56
2021-11-30	0.19	0.31	0.37	0.43	0.55
2021-12-01	0.21	0.31	0.36	0.41	0.52
2021-12-02	0.13	0.22	0.27	0.32	0.40
2021-12-03	0.30	0.39	0.43	0.48	0.57
2021-12-04	0.04	0.12	0.17	0.21	0.29
2021-12-05	0.27	0.34	0.37	0.41	0.48
2021-12-06	0.26	0.32	0.35	0.38	0.44
2021-12-07	0.15	0.21	0.24	0.27	0.32
2021-12-08	0.46	0.51	0.53	0.56	0.61
2021-12-09	0.63	0.67	0.69	0.72	0.76

Table S2. The estimated additive difference in growth rate when daily cases is identical as JHU daily report (factor = 1).

	**2.50%**	**25%**	**50%**	**75%**	**97.50%**
2021-11-28	0.21	0.37	0.45	0.54	0.73
2021-11-29	0.16	0.29	0.36	0.43	0.56
2021-11-30	0.19	0.31	0.37	0.43	0.55

2021-12-01	0.21	0.31	0.36	0.41	0.52
2021-12-02	0.13	0.22	0.27	0.32	0.40
2021-12-03	0.30	0.39	0.43	0.48	0.57
2021-12-04	0.04	0.12	0.17	0.21	0.29
2021-12-05	0.27	0.34	0.37	0.41	0.48
2021-12-06	0.26	0.32	0.35	0.38	0.44
2021-12-07	0.15	0.21	0.24	0.27	0.32
2021-12-08	0.46	0.51	0.53	0.56	0.61
2021-12-09	0.63	0.67	0.69	0.72	0.76

Table S3. The estimated additive difference in growth rate when daily cases is 2-fold as JHU dai-ly report (factor = 2).

	**2.50%**	**25%**	**50%**	**75%**	**97.50%**
2021-11-28	0.28	0.42	0.49	0.56	0.72
2021-11-29	0.17	0.29	0.34	0.40	0.51
2021-11-30	0.22	0.32	0.37	0.42	0.52
2021-12-01	0.25	0.32	0.37	0.41	0.49
2021-12-02	0.14	0.21	0.25	0.29	0.36
2021-12-03	0.36	0.42	0.46	0.49	0.56
2021-12-04	0.05	0.11	0.14	0.18	0.24
2021-12-05	0.30	0.36	0.38	0.41	0.46
2021-12-06	0.29	0.33	0.36	0.38	0.42
2021-12-07	0.17	0.21	0.23	0.25	0.29
2021-12-08	0.48	0.52	0.54	0.55	0.59
2021-12-09	0.65	0.68	0.70	0.71	0.74

References

1. Hale, T.; Angrist, N.; Goldszmidt, R.; Kira, B.; Petherick, A.; Phillips, T.; Webster, S.; Cameron-Blake, E.; Hallas, L.; Majumdar, S.; et al. A global panel database of pandemic policies (Oxford COVID-19 Government Response Tracker). *Nat. Hum. Behav* .**2021**, *5*, 529–538.

2. Ritchie, H.; Mathieu, E.; Rodes-Guirao, L.; Appel, C.; Giattino, C.; Ortiz-Ospina, E.; Hasell, J.; Macdonald, B.; Dattani, S.; Roser, M. Coronavirus Pandemic (COVID-19). Available online: https://ourworldindata.org/coronavirus (accessed on 1 December 2021).

3. Suchard, M.A.; Lemey, P.; Baele, G.; Ayres, D.L.; Drummond, A.J.; Rambaut, A. Bayesian phylogenetic and phylodynamic data integration using BEAST 1.10. *Virus Evol.* **2018**, *4*, vey016.

4. Gill, M.S.; Lemey, P.; Faria, N.R.; Rambaut, A.; Shapiro, B.; Suchard, M.A. Improving Bayesian population dynamics inference: A coalescent-based model for multiple loci. *Mol. Biol. Evol.* **2013**, *30*, 713–724.

5. Volz, E.; Mishra, S.; Chand, M.; Barrett, J.C.; Johnson, R.; Geildergerg, R.; Hinsley, W.R.; Laydon, D.R.; Dabbrera, G.; O'Toole, A.; et al. Assessing transmissibility of SARS-CoV-2 lineage B.1.1.7 in England. *Nature* **2021**, *593*, 266–269.

6. Guner, R.; Hasanoglu, I.; Aktas, F. COVID-19: Prevention and control measures in community. *Turk J. Med. Sci.* **2020**, *50*, 571–577.
